# Risk Factor Analysis of CRE Infections at Different Anatomical Sites in ICU Patients

**DOI:** 10.3390/antibiotics14090884

**Published:** 2025-09-01

**Authors:** Guoxing Tang, Huijuan Song, Liyan Mao, Shaozhen Yan, Lei Tian, Cui Jian, Zhongju Chen, Ziyong Sun, Yue Wang

**Affiliations:** Department of Laboratory Medicine, Tongji Hospital, Tongji Medical College, Huazhong University of Science and Technology, Wuhan 430030, China; guoxingtang@tjh.tjmu.edu.cn (G.T.); huijuansong@tjh.tjmu.edu.cn (H.S.); liyanmao@tjh.tjmu.edu.cn (L.M.); yansz@tjh.tjmu.edu.cn (S.Y.); leitian@tjh.tjmu.edu.cn (L.T.); jiancui@tjh.tjmu.edu.cn (C.J.); chenzhongjutj@tjh.tjmu.edu.cn (Z.C.)

**Keywords:** carbapenem-resistant Enterobacteriaceae, risk factor, respiratory tract infection, urinary tract infection, bloodstream infection

## Abstract

**Objectives**: This study aimed to identify differences in risk factors for carbapenem-resistant Enterobacteriaceae (CRE) infections across different anatomical sites and to explore risk factors associated with mortality in CRE-infected patients. **Methods**: Patients who underwent CRE screening and were subsequently diagnosed with CRE infections were included and categorized by infection site: respiratory tract (RTI), urinary tract (UTI), and bloodstream (BSI). Forty ICU patients without CRE infection were randomly selected as controls. Statistical comparisons were performed using the Mann–Whitney U or Chi-square test, as appropriate. Potential risk factors were evaluated via univariate and multivariate analyses, and a predictive model was constructed, with its performance assessed using ROC curve analysis. **Results**: CRE colonization was identified as a common independent risk factor across all three groups (RTI, UTI, and BSI). Infection-site-specific analyses revealed independent risk factors: RTI was associated with mechanical ventilation, UTI with trauma, and BSI with gastrointestinal injury. Predictive models for RTI, UTI, and BSI demonstrated good discrimination, with ROC AUCs of 0.94, 0.94, and 0.95, respectively. In the analysis of Survived versus Deceased patients, the BSI group had the highest mortality, though the difference was not statistically significant. Deceased patients exhibited significantly higher PCT levels than Survived patients (*p* = 0.005). Prior use of carbapenems and antifungal agents, as well as Ln(PCT), were independently associated with mortality in CRE-infected patients. **Conclusions**: Risk factors for CRE infections vary across anatomical sites, with CRE colonization, mechanical ventilation, trauma, and gastrointestinal injury playing key roles. Overuse of antibiotics and elevated inflammatory responses are associated with increased mortality. These findings provide evidence for early identification of high-risk patients and optimization of individualized treatment strategies.

## 1. Introduction

The detection rate of carbapenem-resistant Enterobacteriaceae (CRE) strains has been steadily rising, presenting a significant public health challenge [1,2,3]. CRE infections are particularly concerning due to their high mortality rates, increased healthcare costs, and limited treatment options [4,5]. The management and control of CRE infections are especially difficult in Intensive Care Unit (ICU) patients [6]. These multidrug-resistant organisms have drawn considerable global attention, with both the World Health Organization (WHO) and the U.S. Centers for Disease Control and Prevention (CDC) classifying CRE as an urgent public health threat [7,8]. As CRE infections continue to spread, there is a pressing need to understand the factors contributing to their spread and persistence.

A growing body of research has investigated potential risk factors for CRE colonization and infection. These factors include prolonged antibiotic use, extended hospitalization, the presence of indwelling medical devices, and immunosuppressive conditions [9,10,11]. Additionally, studies have highlighted that certain patient populations—such as those with liver disease, hematological disorders, or those undergoing organ transplantation—may be particularly vulnerable to CRE infections [10,12,13]. This suggests that the risk factors for CRE infection may vary considerably depending on a patient’s underlying health conditions and exposure to healthcare environments.

Among the various clinical manifestations of CRE infections, bloodstream infections (BSIs) are of particular concern due to their high severity, challenging treatment options, and significant impact on patient outcomes [4,14]. Despite this, much of the research surrounding CRE infections has focused primarily on CRE BSI, while the risk factors and outcomes of CRE infections in other infection sites have received relatively less attention. The lack of comprehensive studies exploring risk factors across different types of CRE infections raises important questions about whether these risk factors differ based on the anatomical site of infection and what the potential implications might be for clinical management.

To address this gap, the aim of this study is to analyze the risk factors for CRE infections in different infection sites, investigate the potential differences in these risk factors, and evaluate the outcomes of CRE infections across different infection sites. The goal is to provide insights that could inform better prevention and treatment strategies for CRE infections.

## 2. Results

### 2.1. The Clinical and Demographic Characteristics of the Participants

A total of 116 patients with CRE infections were included in this study, comprising 68 in the respiratory tract infection (RTI) group, 20 in the urinary tract infection (UTI) group, and 28 in the BSI group. There were no statistically significant differences in age and gender among the three groups of patients. Additionally, 40 age- and gender-matched patients who underwent CRE screening but did not develop a CRE infection were selected as the control group. The clinical and demographic characteristics of the participants are described in Table 1. Among all CRE-infected patients, the majority of isolates were carbapenem-resistant *Klebsiella pneumoniae* (CRKPN) (92.11%), while smaller proportions included carbapenem-resistant *Escherichia coli* (CRECO), *Enterobacter cloacae*, *Serratia marcescens*, and *Klebsiella aerogenes*. Among CRE strains, those isolated from the UTI group exhibited a significantly higher resistance rate to ceftazidime/avibactam (CZA) compared to those from the RTI and BSI groups (*p* < 0.05). Additionally, the RTI group showed a higher resistance rate to polymyxin B than the BSI group (Table 1).

### 2.2. Laboratory Findings and Clinical Features of CRE-Infected Patients in Different Anatomical Sites

**Figure 1:**
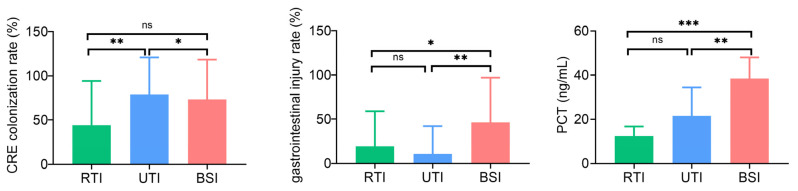
Representative differences in clinical and laboratory features among RTI, UTI, and BSI patients. Bar chart showing the CRE colonization rates, gastrointestinal injury rates, and PCT levels in RTI, UTI, and BSI groups. Data are presented as mean and SD. * *p* < 0.05; ** *p* < 0.01; *** *p* < 0.001; ns, not significant.

The risk factors for CRE infection across different anatomical sites exhibit both similarities and differences. The RTI group exhibited significantly higher rates of CRE colonization, mechanical ventilation, endotracheal intubation, central venous catheter placement, and trauma compared to the control group. Additionally, compared to the control group, the RTI group had significantly higher levels of hypersensitive C-reactive protein (hsCRP) and neutrophils (%) (Table 2). In the UTI group, the CRE colonization rate, endotracheal intubation rate, and trauma rate, as well as hsCRP levels were significantly higher than the control group, with no significant differences observed in other clinical features and laboratory indicators (Table 2). In the BSI group, the CRE colonization rate, the use of mechanical ventilation, the endotracheal intubation rate, and the gastrointestinal injury rate were significantly higher than those in the control group. In the BSI group, prior use of enzyme-inhibitor antibiotics was significantly lower, whereas procalcitonin (PCT), hsCRP, and NEU (%) levels were significantly higher compared to the control group (Table 2). Among different anatomical sites, the UTI group showed a significantly higher CRE colonization rate than the other two groups, while the BSI group exhibited significantly higher rates of gastrointestinal injury and elevated PCT levels compared to both the RTI and UTI groups (Table S1, Figure 1).

### 2.3. Risk Factors for CRE Infection and Predictive Model Construction in Patients with RTI, UTI, and BSI

**Figure 2:**
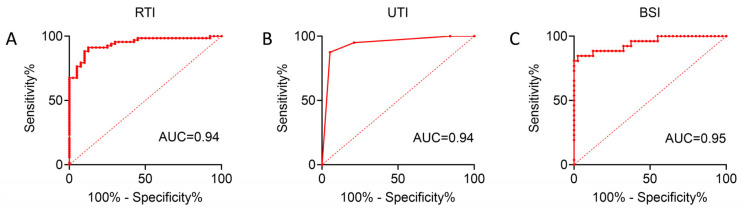
ROC curves of logistic regression models evaluating the predictive performance for CRE infection in RTI/UTI and BSI groups (**A**–**C**). The AUC values are shown, indicating the discriminative ability of each model.

To further clarify the risk factors for CRE infection at different anatomical sites, univariate and multivariate logistic regression analyses were performed on the indicators with statistical significance in Table 2 (hsCRP was excluded due to substantial missing data), and predictive models were subsequently established to distinguish infection groups from controls. In the RTI group, univariate analysis suggested that CRE colonization, hepatic insufficiency, ventilator therapy, trauma, and neutrophils (%) were associated with CRE infection, while multivariate analysis identified CRE colonization, ventilator therapy, trauma, and neutrophils (%) as variables in the final model, among which CRE colonization, ventilator therapy, and neutrophils (%) were independent risk factors. In the UTI group, CRE colonization, hepatic insufficiency, tracheal intubation, and trauma were associated with CRE infection, and multivariate analysis demonstrated that CRE colonization and trauma were independent risk factors retained in the final model. In the BSI group, univariate analysis revealed associations with CRE colonization, diabetes, ventilator therapy, gastrointestinal injury, and neutrophils (%), and multivariate analysis indicated that CRE colonization, ventilator therapy, and neutrophils (%) entered the final model, with CRE colonization being an independent risk factor. The receiver operating characteristic (ROC) curves derived from the three models yielded areas under the curve (AUCs) of 0.95, 0.94, and 0.94, respectively (Table 3, Table 4 and Table 5, Figure 2A–C), indicating good discriminatory and predictive performance across different anatomical sites.

### 2.4. Clinical Characteristics and Mortality Risk Factors in Patients with CRE Infections

**Figure 3:**
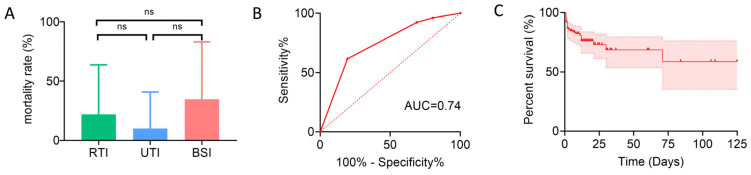
Mortality and prognostic analyses in patients with CRE infections. (**A**) Mortality rates among patients with RTI, UTI, and BSI, shown as bar charts. ns, not significant. (**B**) ROC curve of the logistic regression model predicting mortality in CRE-infected patients, with the AUC indicating discriminative performance. (**C**) Kaplan–Meier survival curve of CRE-infected patients based on in-hospital mortality (censored at discharge), with 95% CI shown.

Mortality rates among patients with CRE infections were 22.06% (15/68) in the RTI group, 10% (2/20) in the UTI group, and 34.62% (9/26) in the BSI group. Despite the highest mortality rate in the BSI group, the difference among the three groups was not statistically significant (Figure 3A). Accordingly, laboratory indicators and clinical characteristics were compared between survivors and non-survivors among all CRE-infected patients. Overall, only a few variables differed significantly between the two groups: non-survivors were more likely to have solid organ malignancies, a higher proportion had received carbapenems and antifungal agents prior to infection, and their PCT levels were significantly elevated, whereas survivors had a higher proportion of trauma cases and more frequent prior use of cephalosporins (Table 6). Univariate and multivariate analyses for mortality showed that solid organ malignancy, trauma, and prior use of carbapenems and antifungal agents were associated with death in univariate analysis; multivariate logistic regression identified trauma and prior carbapenem use as independent variables in the final model, with carbapenem use being an independent risk factor. The ROC curve of this model yielded an AUC of 0.74, indicating moderate discriminative ability (Table 7, Figure 3B). Considering the skewed distribution and extreme values of PCT, natural logarithmic transformation [Ln(PCT)] was applied, followed by repeated univariate and multivariate analyses; after transformation, PCT was incorporated into the final model as an independent predictor of mortality, although the overall discriminative performance was only slightly improved (AUC = 0.75) (Table S2, Figure S1). Kaplan–Meier survival analysis showed that overall survival gradually declined over the course of hospitalization; as only in-hospital mortality was assessed, censoring occurred in the survival curve (Figure 3C). These findings indicate a generally poor prognosis for patients with CRE infection during the hospital stay.

## 3. Discussion

ICU patients face a higher risk of CRE infections and treatment failure due to immunosuppression, prolonged hospitalization, and frequent antibiotic use, posing significant challenges to their clinical management [15,16,17]. However, the severity of CRE infections varies by anatomical site, and the associated risk factors differ accordingly. This study primarily focused on comparing the differences in risk factors for CRE infections at different anatomical sites and exploring risk factors for mortality in CRE-infected patients.

Previous studies have identified several risk factors for CRE infections, including CRE colonization, mechanical ventilation, prolonged hospitalization, immunosuppressive conditions, and long-term antibiotic use [18,19,20]. Through univariate, multivariate, and logistic regression analyses, we systematically evaluated the risk factors and their predictive value. We identified CRE colonization as a common risk factor across all three groups, highlighting its central role in the pathogenesis of CRE infections. In addition, our study revealed distinct site-specific risk factors: mechanical ventilation in the RTI group, trauma in the UTI group, and gastrointestinal injury in the BSI group. Although some of these factors have been reported previously [21,22,23], our study provides the systematic comparison across different infection sites, underscoring the unique risk patterns of CRE infections by anatomical location. These findings also suggest potential differences in the sources or transmission pathways of CRE across sites. Furthermore, ROC curve analysis demonstrated good discriminatory ability, supporting its clinical utility in the early identification of high-risk patients.

Furthermore, patients with BSI caused by CRE exhibited significantly higher levels of inflammatory markers than those in the other groups. This finding suggests a more pronounced systemic inflammatory response in BSI cases, which may contribute to the increased severity and mortality observed in this group. As previously reported, BSI caused by CRE has received widespread attention due to its high mortality rate and severe clinical consequences [24,25,26]. In our study, the BSI group had the highest mortality rate; however, the difference was not statistically significant compared to the RTI and UTI groups. This may be attributed to the relatively small sample size, which could have limited the statistical power and affected the significance of our findings. Interestingly, although CRE-related BSI are particularly concerning, our study found that CRE strains in the BSI group had the lowest resistance rates to CZA and polymyxin B among the three infection groups. In recent years, the emergence of CRE strains resistant to both CZA and polymyxin B has become an increasingly concerning issue [27,28]. This observation has not been widely reported in previous studies and may reflect potential differences in resistance characteristics among CRE strains from different infection sites. Possible explanations for this finding include variations in host immune status, selective pressures specific to the bloodstream environment, and differences in prior antibiotic exposures among patients. It is also possible that these patterns are influenced by local epidemiological factors and may not be generalizable to other regions. Given the limited sample size and the single-center nature of our study, further multicenter studies with larger cohorts are needed to validate these findings and to explore the underlying biological and epidemiological mechanisms.

Moreover, previous studies have reported that immunosuppressive conditions, such as malignancies and organ transplantation, as well as antibiotic use, are associated with increased mortality in CRE-infected patients [10,29,30,31]. Our study supports this trend, univariate and multivariate analyses of survivors and non-survivors revealed that prior use of carbapenems and antifungal agents was independently associated with increased mortality, consistent with previous findings [29,32]. Interestingly, trauma was more frequent in survivors than in non-survivors, suggesting that while trauma may predispose to CRE infection, it is not a direct determinant of mortality. We further observed significantly higher PCT levels in the non-survivor group. Although the difference was not statistically significant in univariate analysis, the log-transformed PCT (Ln[PCT]) was retained in the final prognostic model. In line with prior studies, this supports the role of excessive inflammatory responses in disease progression and death, where marked elevations in inflammatory markers serve as a warning signal. Overall, prudent antibiotic use and timely control of infection-associated inflammation are crucial for improving patient outcomes.

This study has several limitations. First, it was conducted in a single center with a limited sample size, which may reduce the robustness of the multivariate models. Second, the lack of statistical significance in mortality differences among the three groups may be attributed to the limited number of patients. Third, although we observed distinctive resistance patterns in CRE strains from BSI patients, these findings may merely reflect local epidemiological characteristics rather than generalizable trends. Fourth, in the Kaplan–Meier survival analysis, data for surviving patients were censored at discharge, as no post-discharge follow-up was performed; this may affect the accuracy of survival estimates. Therefore, larger multicenter studies with greater statistical power are needed to validate and expand upon our observations.

## 4. Materials and Methods

### 4.1. Study Subjects

Patients admitted to the ICU of Tongji Hospital, Tongji Medical College, Huazhong University of Science and Technology, between January 2021 and September 2023, who voluntarily underwent CRE screening and subsequently contracted CRE were included in this study. Based on the anatomical site of infection, these patients were categorized into three groups: RTI, UTI, and BSI. Additionally, a control group of 40 ICU patients who underwent voluntary CRE screening during the same period but did not develop a CRE infection was randomly selected. The control group was set at twice the size of the group with the fewest cases among the three groups to balance statistical power across groups. Furthermore, CRE-infected patients were classified into Survived and Deceased groups according to in-hospital mortality (up to the point of discharge). This study was approved by the Ethics Committee of Tongji Medical College, Huazhong University of Science and Technology, Wuhan, China (ID: 2021-S013).

### 4.2. Design and Definition

Each patient provided a perirectal swab to assess for the presence of CRE colonization. The collected rectal swabs were immediately inoculated onto a chromogenic agar plate containing carbapenem as a selective agent (CHROMagar, La Plaine Saint-Denis, France) for CRE screening. All isolated bacterial strains were identified using a MALDI-TOF mass spectrometer (Bruker Daltonics, San Jose, CA, USA) for accurate species identification to ensure accurate species identification. The antimicrobial susceptibility of the isolates, specifically to meropenem and imipenem, was determined using the Kirby–Bauer disk diffusion method. The results were interpreted according to the Clinical and Laboratory Standards Institute (CLSI) M100-ED30 [33] guidelines for breakpoint determination, ensuring standardized and reliable susceptibili assessment.

### 4.3. Data Collection

The data were collected retrospectively from electronic medical records, mainly including variables potentially related to CRE infection. These variables encompassed general information (gender, age, department), underlying conditions (such as hypertension, diabetes, solid organ tumors, hematological malignancies, impaired immune function, gastrointestinal injury), invasive procedures and devices (hematopoietic stem cell transplantation, surgery, mechanical ventilation, central venous catheter, urinary catheter, gastric tube, drainage tube), antibiotic exposure (defined as the use of the specific antibiotic during the period from hospital admission to the occurrence of CRE infection), and routine laboratory data (including WBC count, neutrophil percentage, PCT, and hsCRP, with laboratory data collected on the day the CRE-positive sample was submitted).

### 4.4. Statistical Analysis

Continuous variables were expressed as mean ± standard deviation (SD) or median (interquartile range, IQR), and comparisons between groups were performed using the Mann–Whitney U test. Categorical variables were expressed as number (%), and group comparisons were conducted using the Chi-square test or Fisher’s exact test. Univariate analyses were first performed to identify potential risk factors associated with outcomes. Variables with *p* < 0.05 in the univariate analysis were then included in a multivariate logistic regression model to determine independent risk factors. ROC curves were plotted based on the established logistic model. KM survival curves were constructed according to patient outcomes. Statistical significance was defined as *p* < 0.05. Statistical analyses were conducted using SPSS version 19.0 (SPSS, Chicago, IL, USA) and GraphPad Prism 8.0 (GraphPad Software, San Diego, CA, USA).

## 5. Conclusions

In summary, this study not only confirmed previously reported risk factors for CRE infections but also systematically compared site-specific risk profiles, highlighting the central role of CRE colonization as well as the independent impact of mechanical ventilation, trauma, and gastrointestinal injury across different infection sites. Moreover, our findings suggest that excessive antibiotic use and hyperinflammatory responses play pivotal roles in patient mortality. These results provide new evidence for understanding the pathogenesis and clinical heterogeneity of CRE infections and may facilitate early risk stratification and the development of individualized therapeutic strategies.

## Figures and Tables

**Table 1:** The demographic and clinical characteristics of the participants.

	RTI (*n* = 68)	UTI (*n* = 20)	BSI (*n* = 26)	Controls (*n* = 40)
Age (years, median (25th–75th percentiles))	60.00 (50.75–68.00)	61.00 (53.00–68.00)	64.00 (52.50–68.75)	60.00 (48.50–72.00)
Males [*n* (%)]	53 (77.94)	13 (68.42)	17 (65.38)	26 (65.00)
Species				
*Klebsiella pneumoniae*	63	17	25	/
*Escherichia coli*	1	1	1	/
*Enterobacter cloacae*	2	2	0	/
*Serratia marcescens*	1	0	0	/
*Klebsiella aerogenes*	1	0	0	/
Antibiotic resistance prevalence [*n* (%)]				
Ceftazidime/Avibactam	10 (14.71)	7 (35.00)	2 (7.69)	
Polymyxin B	4 (5.88)	/*	0 (0.00)	

RTI, respiratory tract infection; UTI, urinary tract infection; BSI, bloodstream infection. Data are presented as number (percentage) or medians (25th–75th centiles). * The urinary tract infection CRE patients did not report sensitivity results for Polymyxin B.

**Table 2:** Clinical data and inflammatory biomarkers in enrolled patients.

	RTI (*n* = 68)	UTI (*n* = 20)	BSI (*n* = 26)	Controls (*n* = 40)	*p* (RTI vs. Controls)	*p* (UTI vs. Controls)	*p* (BSI vs. Controls)
Colonization	30 (44.12)	15 (78.95)	19 (73.08)	2 (5.00)	<0.001	<0.001	<0.001
Diabetes	14 (20.59)	4 (21.05)	9 (34.62)	5 (12.50)	0.286	0.393	0.032
Hypertension	33 (48.53)	7 (36.84)	14 (53.85)	14 (35.00)	0.171	0.890	0.130
Cardiopathy	8 (11.76)	4 (21.05)	6 (23.08)	9 (22.50)	0.139	0.900	0.956
Hypoproteinemia	33 (48.53)	11 (57.89)	14 (53.85)	16 (40.00)	0.390	0.197	0.270
Serosal cavity effusion	29 (42.65)	6 (31.58)	10 (38.46)	14 (35.00)	0.433	0.795	0.775
Septic shock	17 (25.00)	5 (26.32)	13 (50.00)	12 (30.00)	0.571	0.770	0.102
Hepatic insufficiency	15 (22.06)	3 (15.79)	8 (30.77)	18 (45.00)	0.012	0.029	0.248
Renal insufficiency	23 (33.82)	8 (42.11)	15 (57.69)	16 (40.00)	0.519	0.878	0.159
Solid organ malignancy	7 (10.29)	2 (10.53)	4 (15.38)	8 (20.00)	0.121	0.142	0.251
Hematological malignancy	2 (2.94)	1 (5.26)	1 (3.85)	3 (7.50)	0.402	0.542	0.356
Maintenance immunosuppressive regimen	2 (2.94)	1 (5.26)	3 (11.54)	4 (10.00)	0.051	0.390	0.907
Ventilator therapy	58 (85.29)	12 (63.16)	22 (84.62)	20 (50.00)	<0.001	0.343	0.004
Mechanical ventilation	68 (100.00)	17 (89.47)	25 (96.15)	40 (100.00)	0.990	0.037	0.211
Tracheal intubation	67 (98.53)	17 (89.47)	22 (84.62)	20 (50.00)	<0.001	0.003	0.004
Central venous catheter	68 (100.00)	18 (94.74)	26 (100.00)	37 (92.50)	0.022	0.749	0.153
Drainage tube	61 (89.71)	16 (84.21)	24 (92.31)	36 (90.00)	0.961	0.521	0.750
Urinary catheter	67 (98.53)	17 (89.47)	25 (96.15)	38 (95.00)	0.281	0.430	0.826
Surgery	25 (36.76)	6 (31.58)	9 (34.62)	10 (25.00)	0.207	0.595	0.399
Trauma	20 (29.41)	6 (31.58)	3 (11.54)	3 (7.50)	0.007	0.016	0.577
Gastrointestinal injury	13 (19.12)	2 (10.53)	12 (46.15)	6 (15.00)	0.587	0.639	0.005
Cephalosporin antibiotics	15 (22.06)	4 (21.05)	3 (11.54)	8 (20.00)	0.801	0.925	0.367
Ceftazidime/avibactam	4 (5.88)	1 (5.26)	1 (3.85)	1 (2.50)	0.419	0.594	0.755
Quinolone antibiotics	23 (33.82)	5 (26.32)	4 (15.38)	14 (35.00)	0.901	0.505	0.080
Aminoglycoside antibiotics	2 (2.94)	2 (10.53)	0 (0.00)	3 (7.50)	0.276	0.697	0.153
Carbapenem antibiotics	29 (42.65)	4 (21.05)	11 (42.31)	17 (42.50)	0.988	0.108	0.988
Tigecycline	10 (14.71)	6 (31.58)	8 (30.77)	6 (15.00)	0.967	0.139	0.126
Polymyxin B	2 (2.94)	3 (15.79)	3 (11.54)	2 (5.00)	0.584	0.164	0.327
Linezolid	11 (16.18)	3 (15.79)	2 (7.69)	5 (12.50)	0.604	0.730	0.535
Glycopeptide antibiotics	9 (13.24)	2 (10.53)	4 (15.38)	7 (17.50)	0.547	0.486	0.822
Antifungal antibiotics	11 (16.18)	4 (21.05)	5 (19.23)	2 (5.00)	0.085	0.057	0.067
PCT	1.63 (0.48–4.55)	1.04 (0.32–5.84)	19.66 (3.72–67.38)	1.59 (0.21–23.42)	0.860	0.956	0.002
hsCRP	137.80 (77.40–235.10)	149.45 (96.13–260.98)	231.95 (147.30–274.00)	44.05 (9.33–123.50)	0.002	0.019	0.001
WBC count	11.53 (8.90–16.61)	11.82 (8.42–16.39)	13.44 (8.15–26.19)	12.44 (8.11–15.69)	0.916	0.770	0.300
Neutrophils (%)	19.24 (10.25–83.08)	12.44 (7.02–72.50)	27.04 (8.05–88.10)	11.03 (7.10–12.89)	<0.001	0.215	0.006

RTI, respiratory tract infection; UTI, urinary tract infection; BSI, bloodstream infection; PCT, procalcitonin; hsCRP, hypersensitive C-reactive protein; WBC, white blood cell. Data are presented as number (percentage) or medians (25th–75th centiles).

**Table 3:** Univariate and multivariate analysis of risk factors for CRE infection in the RTI group.

	Univariate Analysis			Multivariate Analysis		
	OR	95% CI	*p*	OR	95% CI	*p*
CRE colonization	15.000	3.346–67.247	<0.001	57.701	4.935–674.607	0.001
Hepatic insufficiency	0.346	0.148–0.807	0.014			
Ventilator therapy	5.800	2.326–14.460	<0.001	36.747	4.276–315.786	0.001
Trauma	5.139	1.419–18.613	0.013	6.381	1.002–40.634	0.050
Neutrophils (%)	1.065	1.023–1.108	0.002	1.097	1.027–1.172	0.006

CRE, carbapenem-resistant enterobacteriaceae; OR, odds ratio; CI, confidence interval.

**Table 4:** Univariate and multivariate analysis of risk factors for CRE infection in the UTI group.

	Univariate Analysis			Multivariate Analysis		
	OR	95% CI	*p*	OR	95% CI	*p*
CRE colonization	71.250	11.783–430.832	<0.001	214.742	18.083–2550.180	<0.001
Hepatic insufficiency	0.229	0.058–0.912	0.037			
Tracheal intubation	8.500	1.732–41.718	0.008			
Trauma	5.592	1.241–26.109	0.025	35.831	2.845–451.201	0.006

**Table 5:** Univariate and multivariate analysis of risk factors for CRE infection in the BSI group.

	Univariate Analysis			Multivariate Analysis		
	OR	95% CI	*p*	OR	95% CI	*p*
CRE colonization	51.571	9.755–272.635	<0.001	94.049	7.386–1197.627	<0.001
Diabetes	3.706	1.075–12.771	0.038			
Ventilator therapy	5.500	1.604–18.864	0.007	20.005	1.271–314.857	0.033
Gastrointestinal injury	4.857	1.521–15.508	0.008			
Neutrophils (%)	1.066	1.018–1.116	0.007	1.114	0.988–1.257	0.079

**Table 6:** Clinical data and inflammatory biomarkers in CRE-infected patients with different outcomes.

	Deceased (*n* = 26)	Survived (*n* = 87)	*p*-Value
Age (years, median (25th–75th percentiles))	57.00 (50.00–67.75)	61.00 (55.00–69.00)	0.368
Males [*n* (%)]	20 (74.07)	63 (72.41)	0.648
Colonization	15 (57.69)	49 (56.32)	0.902
Diabetes	4 (15.38)	23 (26.44)	0.246
Hypertension	10 (38.46)	44 (50.57)	0.278
Cardiopathy	3 (11.54)	15 (17.24)	0.486
Hypoproteinemia	11 (42.31)	47 (54.02)	0.294
Serosal cavity effusion	11 (42.31)	34 (39.08)	0.768
Septic shock	7 (26.92)	28 (32.18)	0.611
Hepatic insufficiency	5 (19.23)	21 (24.14)	0.602
Renal insufficiency	13 (50.00)	33 (37.93)	0.272
Solid organ malignancy	6 (23.08)	7 (8.05)	0.035
Hematological malignancy	2 (7.69)	1 (1.15)	0.069
Maintenance immunosuppressive regimen	3 (11.54)	3 (3.45)	0.106
Ventilator therapy	22 (85.62)	70 (80.46)	0.633
Mechanical ventilation	26 (100)	84 (96.55)	0.337
Tracheal intubation	24 (92.31)	82 (94.25)	0.718
Central venous catheter	26 (100)	86 (98.85)	0.583
Drainage tube	24 (92.31)	77 (88.51)	0.581
Urinary catheter	25 (96.15)	84 (96.55)	0.923
Surgery	7 (26.92)	33 (37.93)	0.303
Trauma	2 (7.69)	27 (21.03)	0.017
Gastrointestinal injury	7 (26.92)	20 (22.99)	0.680
Cephalosporin antibiotics	1 (3.85)	21 (24.14)	0.022
Ceftazidime/avibactam	1 (3.84)	5 (5.74)	0.704
Quinolone antibiotics	10 (38.46)	22 (25.29)	0.191
Aminoglycoside antibiotics	0 (0.00)	4 (4.60)	0.266
Carbapenem antibiotics	17 (65.38)	27 (31.03)	0.002
Tigecycline	8 (30.77)	16 (18.39)	0.176
Polymyxin B	3 (11.54)	5 (5.74)	0.312
Linezolid	2 (7.69)	14 (16.19)	0.281
Glycopeptide antibiotics	5 (19.23)	10 (11.49)	0.308
Antifungal antibiotics	9 (34.62)	11 (12.64)	0.010
PCT	13.00 (2.93–18.28)	1.63 (0.43–7.61)	0.005
hsCRP	137.80 (97.20–196.60)	160.25 (110.93–268.90)	0.890
WBC count	17.28 (8.42–20.53)	11.46 (8.61–15.87)	0.219
Neutrophils (%)	18.96 (7.69–85.38)	19.01 (9.29–83.30)	0.830

CRE, carbapenem-resistant Enterobacteriaceae; PCT, procalcitonin; hsCRP, hypersensitive C-reactive protein; WBC, white blood cell. Data are presented as number (percentage) or medians (25th–75th centiles).

**Table 7:** Univariate and multivariate analysis of risk factors for mortality in patients with CRE infection.

	Univariate Analysis			Multivariate Analysis		
	OR	95% CI	*p*	OR	95% CI	*p*
Solid organ malignancy	3.429	1.037–11.332	0.043			
Trauma	0.185	0.041–0.840	0.029	0.167	0.035–0.787	0.024
Carbapenem antibiotics	4.198	1.661–10.605	0.002	4.516	1.731–11.786	0.002
Antifungal antibiotics	3.658	1.311–10.204	0.013			

## Data Availability

The data supporting this study’s findings are available from the corresponding author (Y.W.) upon reasonable request and with permission of the Tongji Hospital (Wuhan, China).

## Supplementary Materials

The following supporting information can be downloaded at: https://www.mdpi.com/article/10.3390/antibiotics14090884/s1, Figure S1: ROC curve of the logistic regression model predicting mortality in CRE-infected patients (with ln[PCT]); Table S1: Clinical data and inflammatory biomarkers in CRE-infected patients at different anatomical sites; Table S2. Univariate and multivariate analysis of risk factors for mortality in patients with CRE infection (with Ln(PCT)).
