# Supplementary figures and images for: Risk Factor Analysis of CRE Infections at Different Anatomical Sites in ICU Patients
Source: Antibiotics (Basel). 2025 Sep 1;14(9):884. doi: 10.3390/antibiotics14090884 (PMC12466705; doi:10.3390/antibiotics14090884)

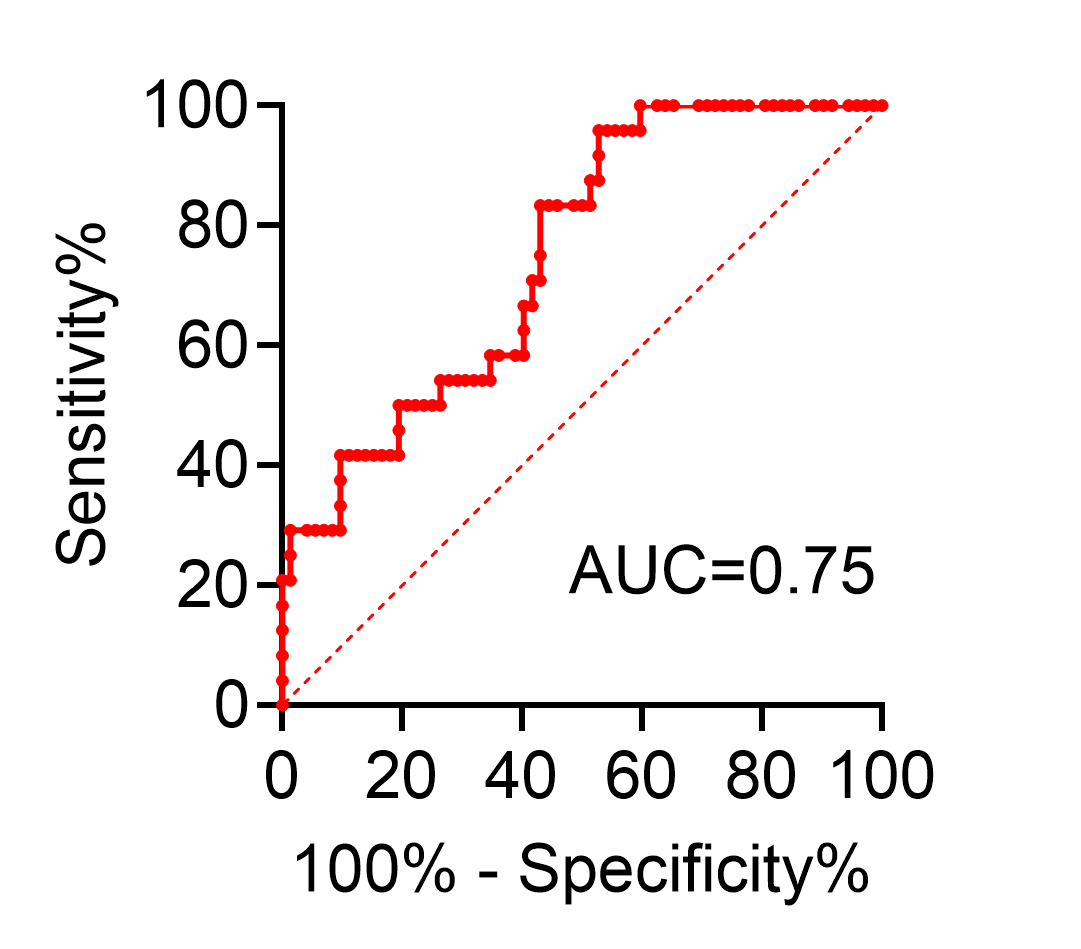

Supplement: Supplementary file 1 [file antibiotics-14-00884-s001.zip › Figure S1.tif]
